# Supplementary figures and images for: The chromosome-scale genome sequence of Triadica sebifera provides insight into fatty acids and anthocyanin biosynthesis
Source: Commun Biol. 2022 Aug 4;5:786. doi: 10.1038/s42003-022-03751-9 (PMC9352727; doi:10.1038/s42003-022-03751-9)

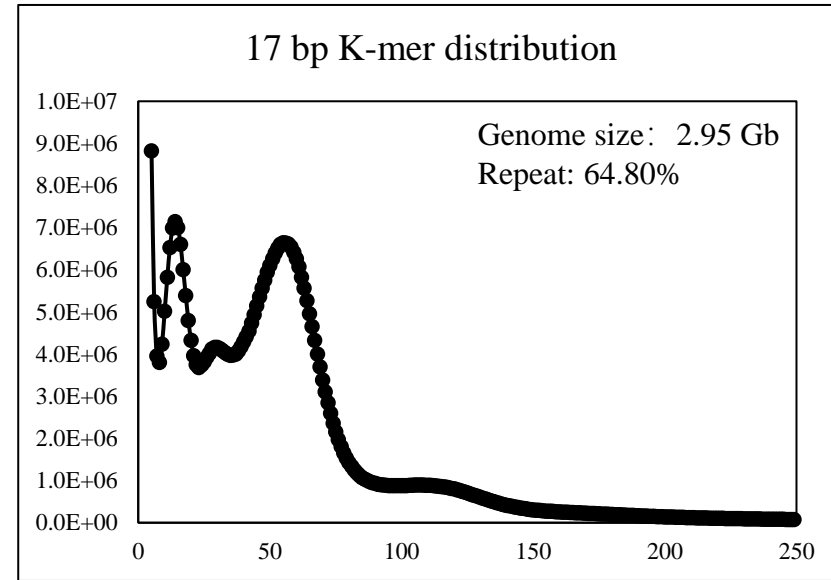

**Supplementary Figure 1.** The 17-bp K-mer distribution of the Chinese tallow tree genome

Supplement: Supplementary file 2 — Supplementary Information [file 42003_2022_3751_MOESM2_ESM.pdf]
