# Supplementary material for: The chromosome-scale genome sequence of Triadica sebifera provides insight into fatty acids and anthocyanin biosynthesis
Source: Commun Biol. 2022 Aug 4;5:786. doi: 10.1038/s42003-022-03751-9 (PMC9352727; doi:10.1038/s42003-022-03751-9)
Supplement: Supplementary file 3 — Description of Additional Supplementary Files [file 42003_2022_3751_MOESM3_ESM.pdf]

Description of Additional Supplementary Files

File name: Supplementary Data 1
Description: Summary of sequence data used for genome assembly.

File name: Supplementary Data 2
Description: Flow cytometry estimation of genome size.

File name: Supplementary Data 3
Description: Information on monoploid, diploid and tetraploid assembly of the Chinese tallow tree.

File name: Supplementary Data 4
Description: Scaffold information of the final monoploid genome of the Chinese tallow tree.

File name: Supplementary Data 5
Description: Summary of mapping rate for 18 RNA-Seq onto the monoploid genome of the Chinese tallow tree.

File name: Supplementary Data 6
Description: Summary of genes annotated with Nr, TAIR, Swiss-Prot, KEGG, and InterPro databases.

File name: Supplementary Data 7
Description: Readable human descriptions of genes in the genome of Chinese tallow trees.

File name: Supplementary Data 8
Description: Cluster comparisons between the Chinese tallow tree diploid and monoploid genomes.

File name: Supplementary Data 9
Description: Species-specific genes in the Chinese tallow tree genome.

File name: Supplementary Data 10
Description: Significantly expanded genes in the genome of the Chinese tallow tree.

File name: Supplementary Data 11
Description: Gene duplication types for the Chinese tallow tree genome.

File name: Supplementary Data 12
Description: Fatty acid-related genes in the genome of the Chinese tallow tree.

File name: Supplementary Data 13
Description: Comparison of the expression of anthocyanin related genes in green and red leaves of Chinese tallow tree.
